# Synergetic effect of mild hypothermia and antioxidant treatment on ROS–mediated neuron injury under oxygen–glucose deprivation investigated by scanning electrochemical microscopy†

**DOI:** 10.1039/d4sc05977h

**Published:** 2024-11-12

**Authors:** Junjie Zhang, Yulin Liu, Yuxiang Zhao, Siyu Zhang, Feng Xu, Fei Li

**Affiliations:** a The Key Laboratory of Biomedical Information Engineering of Ministry of Education, School of Life Science and Technology, Xi'an Jiaotong University Xi'an 710049 P. R. China feili@mail.xjtu.edu.cn; b Bioinspired Engineering and Biomechanics Center (BEBC), Xi'an Jiaotong University Xi'an 710049 P. R. China

## Abstract

Ischemic stroke and reperfusion injury result in neuronal damage and dysfunction associated with oxidative stress, leading to overproduction of cellular reactive oxygen species (ROS) and reactive nitrogen species (RNS). *In situ* monitoring of the transient ROS and RNS effluxes during rapid pathologic processes is crucial for understanding the relationship between progression of cell damage and role of oxidative stress, and developing the corresponding neuroprotective strategies. Herein, we built oxygen glucose deprivation (OGD) and mild hypothermic (MH) models to mimic the *in vitro* conditions of ischemic stroke and MH treatment. We used scanning electrochemical microscopy (SECM) to *in situ* monitor H_2_O_2_ and nitric oxide (NO) effluxes from HT22 cells under the OGD and MH treatment conditions. Through quantitative analysis of the H_2_O_2_ and NO efflux results, we found that the cellular oxidative stress was primarily manifested through ROS release under OGD conditions, and the MH treatment partially suppressed the excessive H_2_O_2_ and NO production induced by reoxygenation. Moreover, the synergistic therapeutic effect of MH with antioxidant treatment significantly reduced the oxidative stress and enhanced the cell survival. Our work reveals the crucial role of oxidative stress in OGD and reperfusion processes, and the effective improvement of cell viability *via* combination of MH with antioxidants, proposing promising therapeutic interventions for ischemic stroke and reperfusion injury.

## Abstract 2

Atmosphere and temperature controlled SECM monitored H_2_O_2_ and NO effluxes of HT22 cells under OGD and MH conditions with antioxidant treatment.

## Graphical abstract

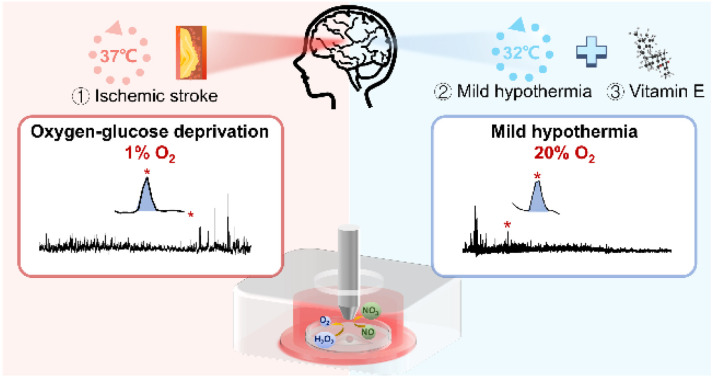

## Introduction

Ischemic stroke as one of the leading causes of global death and disability can rapidly induce neuronal apoptosis due to oxygen deprivation and adenosine triphosphate (ATP) depletion caused by blockage of cerebral blood vessels.^1,2^ Immediate intervention to restore blood flow and salvage neuronal cells is crucial for effective reperfusion.^3^ However, the process of ischemia and the subsequent reperfusion can lead to extensive free radical production and inflammatory responses that exacerbate cellular damage.^4^ Mild hypothermia (MH), an intervention to reduce the body's core temperature to a range between 32 °C and 35 °C, has been proven to be beneficial for various brain injuries (*e.g.*, stroke and traumatic brain injury), through attenuating cellular metabolic demands, inhibiting the inflammatory response and activating multiple signaling pathways (*e.g.*, IGF-1R/AKT and IRAK2/NF-κB pathways).^5–7^ To better understand the underlying mechanisms of ischemia-reperfusion injury and devise effective prevention and treatment strategies, oxygen–glucose deprivation (OGD) and mild hypothermic culture models have been developed and used to stimulate the pathophysiological processes of cerebral ischemic injury and hypothermia therapy *in vitro*.^8–10^

During OGD and reperfusion, intracellular pathways of generation of reactive oxygen species (ROS) and reactive nitrogen species (RNS) are over-activated because of the impaired energy metabolism and subsequent reoxygenation.^11^ Meanwhile, the reduced synthesis or activity of intercellular endogenous antioxidant defense systems impairs the elimination of accumulation of ROS and RNS, which triggers cellular damage (*e.g.*, lipid peroxidation and organelle dysfunction), ultimately causing neuronal death and brain tissue injury.^12–15^ Thus, investigation of the dynamics of oxidative stress levels of ROS and RNS of neurons during OGD and reperfusion is crucial for understanding the role of oxidative stress in neuronal injury and the mechanism of ischemic stroke. The conventional methods for measuring cellular ROS and RNS, such as fluorescent staining and spectrophotometry methods, need labelling of cells with specific optical probes and are limited by their long incubation and relatively low quantitative accuracy,^16,17^ which cannot meet the need of monitoring the rapid progression of the ischemia-reperfusion injury process. Electrochemical methods which are label-free, with high sensitivity and real-time monitoring capabilities, are suitable for noninvasive and quantitative detections of ROS and RNS effluxes. Scanning electrochemical microscopy (SECM) is an electrochemical scanning probe microscope using a μm nm^−1^-sized electrode as its probe, with the capability to precisely monitor the chemical species released from cells and track the dynamic interfacial processes across the cell membrane through recording faradaic/ion current and potential changes from various charge transfer reactions around cells.^18–20^ In previous reports, SECM has been applied to *in situ* characterize the dynamics of ROS and RNS molecules of single living cells in a noninvasive manner,^21–23^ such as by monitoring the cellular released H_2_O_2_ and NO and monitoring the dynamics of intracellular ROS and RNS (including H_2_O_2_, ONOO^−^, NO˙ and NO_2_^−^) molecules from several cell lines.^24–26^ Thus, SECM can be an ideal tool for monitoring the ROS/RNS effluxes of neurons during OGD and MH treatment.

**Scheme 1:**
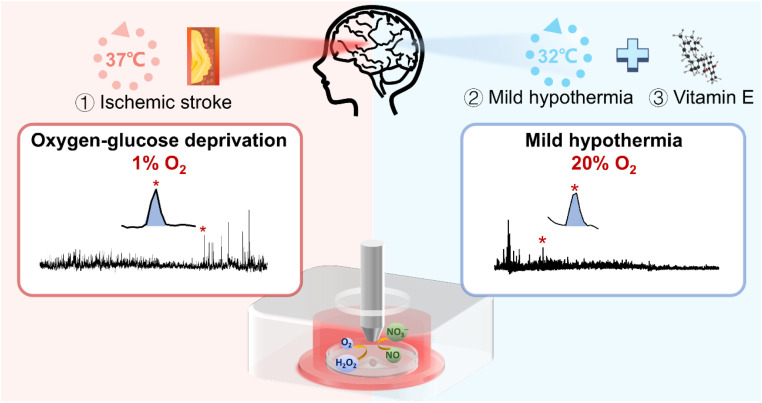
Schematic diagram of application of the SECM platform to *in situ* monitor H_2_O_2_ and NO effluxes of HT22 cells under OGD and MH treatments.

In this work, we investigated the role of oxidative stress and its underlying mechanisms of neuronal injury under ischemic stroke, as well as the synergistic therapeutic effect of physical mild hypothermia combined with chemical drug intervention (Scheme 1). First, to mimic the *in vitro* conditions of ischemic stroke and MH, we constructed the OGD and MH cell models by selecting HT22 cells, an immortalized mouse hippocampal cell line, as the OGD and reperfusion model representative and culturing them under atmosphere- and temperature-controlled experimental conditions. We determined the durations of OGD and MH processes of HT22 cells *via* colorimetric and fluorescence characterization studies of cell viability and intracellular ROS and NO levels. Then we employed SECM to *in situ* monitor the frequencies and amounts of H_2_O_2_ and NO effluxes of HT22 cells under OGD and MH treatment alone or in combination with antioxidant (vitamin E in this case) intervention. The results showed that the dramatic release of H_2_O_2_ and NO during OGD were eliminated by both the MH treatment alone and the MH combined with vitamin E treatment, and the synergetic effect of MH with antioxidant intervention had the highest efficiency for reducing the oxidative stress level of HT22 cells suffering from OGD and markedly improved the cell viability. Our work contributes to a better understanding of the role of oxidative stress in neuronal injury caused by OGD and the potential therapeutic strategy of MH combined with antioxidants for OGD.

## Results and discussion

### Oxidative stress of HT22 cells under OGD and MH treatment

**Fig. 1:**
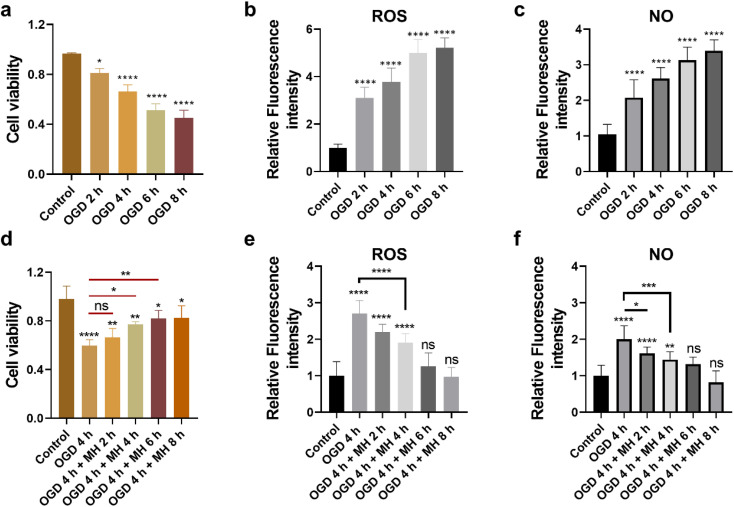
(a and d) Colorimetric and (b, c, e and f) fluorescence characterization results of cellular viability and intracellular ROS and NO levels of HT22 cells under OGD and MH conditions. (a) Viability and (b and c) intracellular ROS and NO levels of HT22 cells after OGD for 0–8 h (*n* = 30). (d) Viability and (e and f) intracellular ROS and NO levels of HT22 cells under OGD for 4 h and following MH treatment for 0–8 h (*n* = 30). All data are depicted as means ± SEM, and graphs were compared by one-way ANOVA (ns, no significant difference, *p < 0.05, **p < 0.01, ***p < 0.001 and ****p < 0.0001).

First, to mimic the ischemic stroke and mild hypothermic treatment conditions, we built *in vitro* cell models using HT22 cells as the ischemic stroke model and then cultured them in glucose-free Earle's balanced salt solution (EBSS) with 1% dissolved oxygen at 37 °C. To investigate the cellular state and oxidative stress level under OGD and MH conditions, we determined the durations of OGD and MH in our experimental system through characterization studies of the three key cellular parameters, *i*.*e*., cell viability, and intracellular ROS and NO levels, under OGD and MH processes using colorimetric and fluorescence methods. From Fig. 1a, we observed that the viability of HT22 cells after the OGD process at 0, 2, 4, 6 and 8 h presented a continuous decrease from 81.2% to 66.1%, 51.3% and 45.2%, respectively, compared to that of the control groups of 96.5% under normal physiological conditions (37 °C, high-glucose Dulbecco's modified eagle medium (HG-DMEM), 5% CO_2_, 20% O_2_ and 75% N_2_). This indicates that the HT22 cells were highly sensitive to glucose and oxygen concentrations of the culture medium and the detrimental effects of reduced oxygen availability and energy deprivation on cell viability. From the fluorescence images and statistical results in Fig. S1a and 1b,† we observed that the intracellular ROS levels of HT22 cells after 2, 4, 6, and 8 h of OGD were 3.1, 3.8, 5.0, and 5.2 times higher than those of the control groups. The intracellular NO levels after 2, 4, 6, and 8 h of OGD were 2.1, 2.6, 3.1, and 3.4 times higher than those of the control groups (Fig. 1c and S1b†). These results show that under 2 h of OGD, the cellular oxidative stress levels significantly increased, while the cell viability remained over 80% compared to that of the control group, indicating that the HT22 cells experienced neuronal oxidative injury and could withstand oxidative stress without succumbing to cell death. Additionally, extending OGD to 4 h resulted in a remarkable decrease in cell viability to ∼60%, indicating that the prolonged OGD duration time caused significant cell death likely due to the increased oxidative injury. Thus, the OGD duration of 4 h was selected as the optimal experimental condition of OGD in the subsequent experiments, which allows the examination of both the onset and progression of neuronal oxidative injury.

Next, we evaluated the therapeutic intervention of MH during reperfusion *via* characterization of the cell viability, and intracellular ROS and NO levels of HT22 cells under MH treatment from 0 to 8 h following 4 h of OGD. The cell viability increased to 66%, 77%, and 82%, and remained above 82% after 2, 4, 6 and 8 h of MH treatment, respectively, displaying a significant difference compared to the OGD group (60%) after 4 h of MH treatment (Fig. 1d). This suggests that the MH intervention can effectively ameliorate the neuronal injury induced by OGD. Moreover, the intracellular ROS and NO levels of HT22 cells of the groups after 4 h of MH treatment continuously decreased and presented a significant difference compared to the OGD groups, indicating that the oxidative stress levels of cells decreased after MH treatment for over 4 h (Fig. 1e, f, S1c and d†), while the intracellular ROS and NO levels of HT22 cells after 6 h of MH treatment did not show significant differences compared to the control groups, indicating that MH can effectively suppress the increase in intracellular ROS levels induced by reoxygenation.^27^ Thus, the MH treatment duration of 4 h was selected as the optimal condition of MH for the following experiments as it can effectively enhance cell viability and reduce oxidative stress in HT22 cells.

### SECM platform for monitoring ROS and NO effluxes of HT22 cells under OGD and MH conditions

**Fig. 2:**
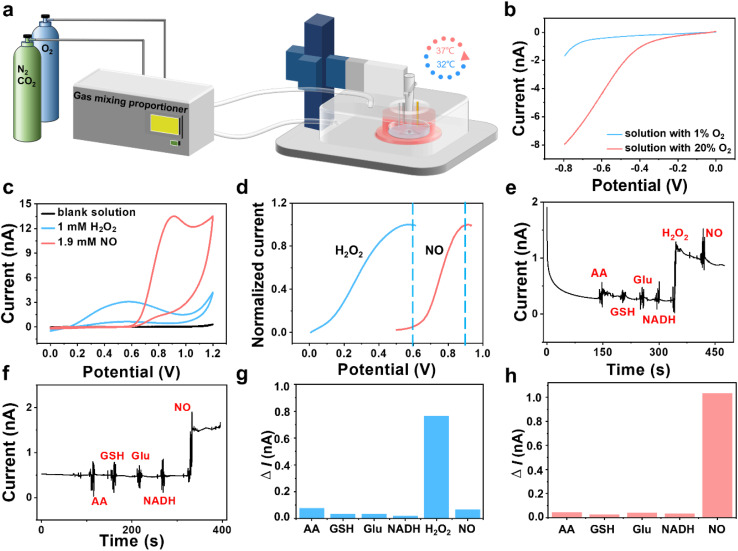
Electrochemical measurements of dissolved oxygen, H_2_O_2_ and NO using an atmosphere- and temperature-controlled SECM platform. (a) Schematic of the atmosphere- and temperature-controlled SECM platform. (b) Linear sweep voltammograms in the advanced Tyrode's solution containing 1% and 20% dissolved oxygen. (c) Cyclic voltammograms in the advanced Tyrode's solution without and with addition of 1 mM H_2_O_2_ and 1.9 mM NO. (d) Normalized oxidation voltammograms of H_2_O_2_ and NO. (e and f) Amperometric responses towards H_2_O_2_ and NO in the presences of interfering chemicals (AA, GSH, Glu and NADH) at 0.6 V and 0.9 V. (g and h) Statistical diagrams of the differences in the current responses in (e and f), respectively.

To *in situ* monitor the H_2_O_2_ and NO effluxes of HT22 cells under OGD and MH conditions, we established an atmosphere- and temperature-controlled SECM platform with integrations of a custom atmospheric chamber to regulate the ratios of O_2_ (1% and 20%) and N_2_ (94% and 75%) and a culturing chamber to incubate cells at 32 and 37 °C (Fig. 2a). From the linear sweep voltammograms in Fig. 2b, we observed that the oxygen reduction currents were significantly reduced in the solution with 1% dissolved oxygen compared to the solution with 20% dissolved oxygen, confirming the oxygen concentration of the culture medium in our SECM platform was under hypoxic conditions. The temperatures in the system can be warmed up to the preset temperature within 5 min and controlled within a narrow range of ±0.2 °C h^−1^ (Fig. S2†). Then we used a three-electrode system with a Pt microelectrode as the working electrode to record cyclic voltammograms in the advanced Tyrode's solution after adding H_2_O_2_ and NO. Distinct oxidation potential peaks were observed at 0.58 V for H_2_O_2_ and 0.9 V for NO with minimal overlap between the two species prior to 0.6 V. And the oxidation current of NO was less than 0.5% of the oxidation current of H_2_O_2_ at 0.6 V, indicating that interference between the oxidation currents of H_2_O_2_ and NO can be negligible in this case (Fig. 2c and d).

Considering the possible interferences from the coexisting redox-active species in cells, such as ascorbic acid (AA), glutathione (GSH), glutamate (Glu) and nicotinamide adenine dinucleotide (NADH),^28,29^ on our electrochemical monitoring of H_2_O_2_ and NO, we used amperometry to evaluate the selectivity of our experimental system. As shown in Fig. 2e, after sequential additions of AA, GSH, Glu, NADH, H_2_O_2_ and NO into the advanced Tyrode's solution, an obvious current response at 0.6 V owing to H_2_O_2_ oxidation was observed, at which potential NO and other coexisting interferents presented low current responses. As shown in Fig. 2f, NO generated a significant oxidation current at 0.9 V with negligible interference from the other coexisting substances. From the histograms of the current differences at 0.6 V and 0.9 V (Fig. 2g and h), we inferred that the current responses of the interferents were less than 10% of the H_2_O_2_ oxidation current at 0.6 V and 5% of the NO oxidation current at 0.9 V, respectively, indicating that the coexisting interferences had a negligible impact on the amperometric detection of H_2_O_2_ and NO in our work.

Next, we recorded the current responses of H_2_O_2_ and NO released from HT22 cells after adding 2,3-dimethoxy-1,4-naphthalenedione (DMNQ) (a ROS inducer) and l-arginine (L-Arg, a substrate for NO generation by neuronal-type nitric oxide synthase (nNOS)). An obvious current response at 0.6 V was recorded after addition of 30 μM DMNQ, indicating rapid H_2_O_2_ production from HT22 cells after adding DMNQ due to the increased extracellular ROS level (Fig. S3a†). Similarly, a rapid increase in the current signal at 0.9 V was observed following addition of 100 μM L-Arg, attributed to the rapid NO production by the intracellular nNOS (Fig. S3b†). These results demonstrate the feasibility of our electrochemical system for monitoring the cellular released H_2_O_2_ and NO.

### Effects of culture temperature and electrode size on electrochemical measurements of cellular released H_2_O_2_ and NO

Based on the Bulter–Volmer equation (eqn (1)) and Strokes–Einstein equation (eqn (2)), the redox currents and the diffusion coefficients of analytes are both affected by the experimental temperature (*i.e.*, OGD (37 °C) and MH (32 °C) in our case).^30^1
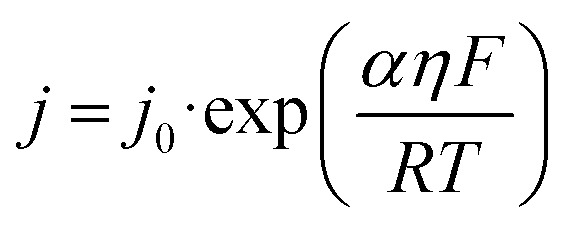
2
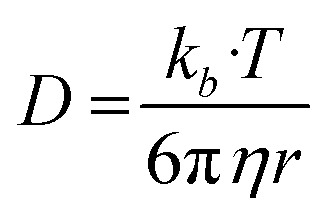
where *j* is the current density, *j*_0_ is the exchange current density, *α* is the kinetic parameter transfer coefficient, *η* is the overvoltage, *F* is Faraday's constant, *R* is the gas constant, *T* is the temperature, *D* is the diffusion coefficient, *k*_b_ is the Boltzmann constant, and *r* is the radius of the diffused particle. In addition, for better monitoring the cell released H_2_O_2_ and NO, the electrode size is also a key parameter since the size of the electrode determines both the closest distance for positioning the electrode to the cell surface and the collection efficiency of cellular released H_2_O_2_ and NO. Thus, before our SECM experiments, we employed the finite element method (FEM) to construct the theoretical models to obtain the concentration maps and amperometric traces of H_2_O_2_ and NO released from the cell membrane at two culture temperatures (32 °C and 37 °C), with three commonly used electrode sizes in the SECM system (10, 25, and 50 μm-in-diameter microdisk electrodes). In our simulations, the electrodes were all set 2 μm above the top of the cell surface (Fig. S4a†).

**Fig. 3:**
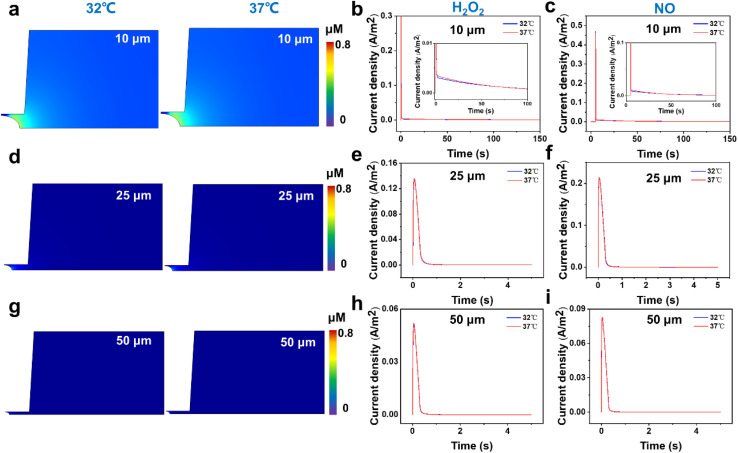
FEM stimulations of (a, d and g) concentration maps of H_2_O_2_ effluxes and (b, c, e, f, h and i) amperometric traces of H_2_O_2_ and NO effluxes released from the cell membrane at 32 °C and 37 °C using (a–c) 10, (d–f) 25 and (g–i) 50 μm-in-diameter microdisk electrodes as the SECM probes. The insets of (b) and (c) are the magnified images of each amperometric trace in the first 100 s in the current density range of 0–0.1 A m^−2^.

First, from the concentration maps and the amperometric traces of H_2_O_2_ and NO effluxes generated by using a 10 μm-in-diameter microdisk electrode in Fig. 3a–c and S4b,† we can see that the H_2_O_2_ and NO effluxes mainly diffuse to the bulk solution rather than being localized in the confined space beneath the microelectrode and the cell membrane, suggesting that the 10 μm-in-diameter microdisk electrode is not large enough to capture the total H_2_O_2_ and NO effluxes. And the simulated amperometric traces of H_2_O_2_ and NO at 37 °C show that the amperometric traces at 37 °C are not well overlapped with the traces at 32 °C. This might be due to the temperature effect on the diffusion coefficients of H_2_O_2_ and NO, causing the differences in the collection efficiency, current responses and mass transports of H_2_O_2_ and NO effluxes at 32 °C and 37 °C (Fig. 3b and c).

To avoid the temperature effect and achieve better collection efficiency of the cellular released H_2_O_2_ and NO, we further analyzed the concentration maps and the amperometric traces of H_2_O_2_ and NO effluxes using 25 and 50 μm-in-diameter microdisk microelectrodes as the SECM probes at 32 °C and 37 °C. As shown in Fig. 3d–f and S4c,† the generated H_2_O_2_ and NO effluxes are confined within the gaps between the 25 μm-in-diameter microdisk electrodes and the cell surfaces, and the simulated amperometric traces of H_2_O_2_ and NO at 37 °C overlap well with the amperometric traces at 32 °C.

These results indicate that the 25 μm-in-diameter electrodes can capture the total released H_2_O_2_ and NO effluxes and the temperature effect on the current responses and the mass transports of H_2_O_2_ and NO can be neglected. Similar results can be observed from the results of concentration maps and amperometric traces of H_2_O_2_ and NO effluxes using a 50 μm-in-diameter microdisk electrode, indicating that a microdisk electrode with a diameter of over 25 μm can collect the cellular released H_2_O_2_ and NO in our experimental system (Fig. 3g–i and S4d†). Considering that the smaller electrode size can minimize the background current and improve signal resolution, the 25 μm-in-diameter microdisk electrode was chosen as the SECM probe to monitor the H_2_O_2_ and NO effluxes from HT22 cells in our subsequent experiments.

### Extracellular ROS and NO effluxes of HT22 cells under OGD *in situ* monitored by SECM

**Fig. 4:**
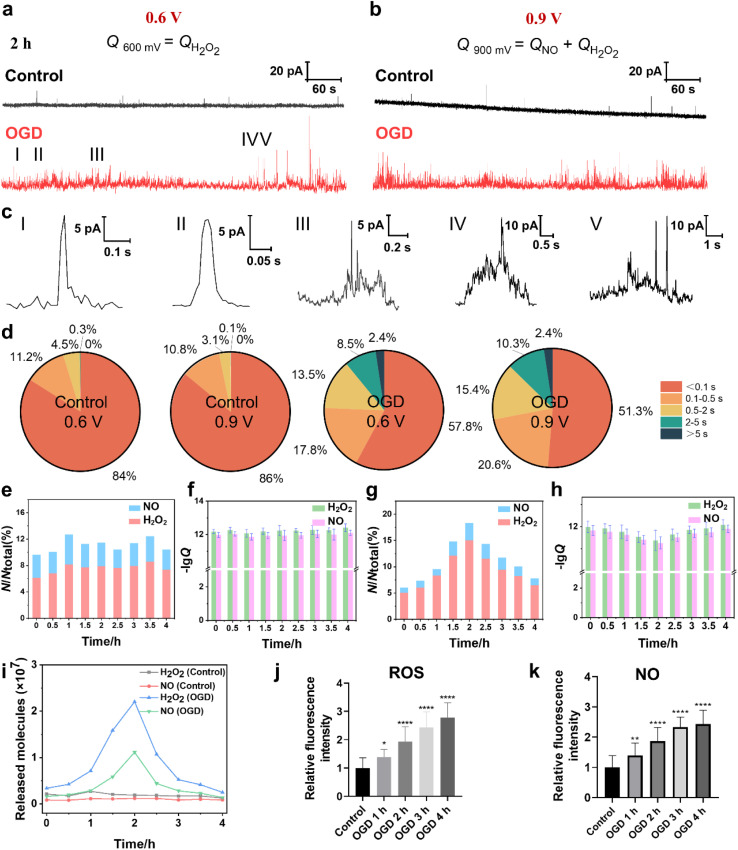
Amperometric traces and H_2_O_2_ and NO effluxes of HT22 cells under normal physiological and OGD conditions. (a and b) Representative amperometric traces of HT22 cells under normal physiological and OGD conditions for 2 h at 0.6 V and 0.9 V. (c) Five different types of amperometric spike shapes featured below amperometric traces. (d) Statistical diagram of the occurrence frequency of different types of amperometric spikes of the control groups and the OGD groups at 0.6 V and 0.9 V. (e) Distributions of the release events of H_2_O_2_ and NO of the control groups detected at 0.6 and 0.9 V *vs.* time. (f) Statistical analyses of the logarithm of *Q* (lg *Q*) of the control groups at 0.6 and 0.9 V during 0–4 h, respectively. (g) The distributions of the release events of H_2_O_2_ and NO of the OGD group detected at 0.6 and 0.9 V *vs.* time. (h) Statistical analyses of lg *Q* of the OGD group at 0.6 and 0.9 V during 0–4 h. (i) Average released molecules of H_2_O_2_ and NO of the control and OGD groups during 0–4 h. (j and k) Statistical analyses of the intracellular H_2_O_2_ and NO effluxes of HT22 cells during 4 h of OGD (*n* = 30). All data are depicted as means ± SEM and graphs were compared by one-way ANOVA (*p < 0.05, **p < 0.01, ***p < 0.001, and ****p < 0.0001).

The cellular ROS and NO levels can significantly increase under OGD and reperfusion, triggering cellular dysfunction and even cell death.^31^ The accumulation of ROS and NO can lead to harmful biochemical reactions inside cells, such as lipid peroxidation of the cell membrane, DNA damage and mitochondrial dysfunction. Thus, we assessed the extent of cellular damage after exposure to OGD and its protective mechanism under MH treatment *via* quantitatively monitoring the extracellular H_2_O_2_ and NO levels using SECM. First, the SECM platform integrated with an adjustable temperature- and atmosphere-controlled chamber described above was used to mimic the normal physiological and OGD conditions. Amperometry was applied to record the oxidative responses of H_2_O_2_ and NO effluxes from HT22 cells under OGD every 30 min for 4 h. As shown in Fig. 4a and b, taking the amperometric traces of HT22 cells under OGD for 2 h as a representative example, a series of distinct individual events of H_2_O_2_ and NO effluxes from HT22 cells at 0.6 V and 0.9 V, respectively, are observed in the OGD groups compared to the control groups. From the categorized spike types based on the duration and the shape features, the most common spikes, identified with simple events, displayed a single maximum peak within 0.5 s (Fig. 4c(I and II)). Meanwhile, the spikes with complex shape features shown in Fig. 4c(III–V), defined as complex events, have similar characteristics to the neurotransmitter exocytosis and ROS events from neurons.^32–34^ In addition, 95.2% and 96.8% of the amperometric spikes at 0.6 V and 0.9 V were simple events under the normal physiological conditions, while the proportions of the simple events obviously decreased to 75.6% and 71.9% under OGD (Fig. 4d), indicating the onset of oxidative stress and cellular injury induced by OGD. Additionally, the statistical frequency distributions of H_2_O_2_ and NO effluxes maintained a consistent ratio of approximately 6.9 : 3.1 over 4 h of incubation under normal physiological conditions, while the ratio shifted dramatically to 8.3 : 1.7 under OGD conditions (Fig. 4e), suggesting that the ROS predominated among oxygen-derived free radicals under OGD-induced stress.

Next, we calculated the charges of the amperometric spikes according to Faraday's law (*Q* = *nzF*, *z*_H_2_O_2__ = 2 and *z*_NO_ = 3 in this case), and obtained the *Q*_0.6V_ and *Q*_0.9V_ by independent time integrations of *i*_0.6V(t)_ and *i*_0.9V(t)_, as well as the number of released molecules (*N*), which was given by the relative *Q*.^35–38^ The averages of *N*_H_2_O_2__ and *N*_NO_ from HT22 cells under normal physiological conditions at 2 h were 0.19 × 10^7^ and 0.12 × 10^7^ molecules, respectively (Fig. 4f). While, during OGD, the HT22 cells exhibited consistent increases of *N*_H_2_O_2__ and *N*_NO_ from 0.34 × 10^7^ and 0.16 × 10^7^ molecules at 0 h, respectively, and reached the maximum values of 2.2 × 10^7^ and 1.1 × 10^7^ molecules at 2 h, respectively. Subsequently, the released *N*_H_2_O_2__ and *N*_NO_ decreased to 0.25 × 10^7^ and 0.14 × 10^7^ molecules at 4 h of OGD (Fig. 4g and i). Moreover, the intracellular ROS levels at 4 h of OGD were 1.38, 1.94, 2.44, and 2.78 times higher than that of the control groups (Fig. 4j and S5a†). And the intracellular NO levels were 1.39, 1.86, 2.33, and 2.43 times higher than that of the control groups (Fig. 4k and S5b†). Both the SECM and fluorescence results show similar trends where the ROS and NO effluxes of HT22 cells rapidly elevated within 2 h of OGD, while the rates of increment subsequently stabilized during 2 to 4 h, revealing that the HT22 cells experienced a significant increase in the oxidative stress level under hypoxia.

A possible reason for the above results might be because the prolonged OGD duration impedes the normal operation of the electron transport chain from the inadequate oxygen and glucose provision.^39^ This disruption diminishes the potential difference between the internal and external sides of the inner mitochondrial membrane, making it easier for leakage to occur during the electron transport process and thus generating a large amount of ROS and inducing the production of NO.^40,41^ Simultaneously, OGD causes a stress response of HT22 cells from an increase in the expression of nNOS and thus the synthesis of a large amount of NO.^42,43^ Furthermore, OGD leads to a decrease in the activities of intracellular antioxidant enzymes (*e.g.*, superoxide dismutase (SOD), catalase (CAT)) and nonenzymatic antioxidants (GSH), which impairs the ability of endogenous antioxidants to scavenge the excessive ROS and promote the accumulation of ROS.^44^ After OGD for 2 h, the ability to generate ROS and NO within cells gradually diminished due to the insufficient amount of oxygen, which manifested as a reduction in the number of ROS release events.

### Extracellular H_2_O_2_ and NO effluxes of HT22 cells under MH combined with vitamin E treatment

According to the above experimental results, we found the rapid generation and release of ROS and NO of HT22 cells induced by OGD, which caused an obvious decrease in cell viability. For further developing the neuroprotective treatment methods, reducing the neuronal oxidative stress of HT22 cells can be one of the strategies. MH, one of the clinically used neuroprotective treatment approaches, can effectively inhibit the production of oxygen-derived free radicals and provide neuroprotection after traumatic brain injury and cerebral ischemia through various molecular mechanisms.^45^ Next, we further explored the therapeutic efficacy of MH through monitoring the H_2_O_2_ and NO effluxes of HT22 cells subjected to 4 h of OGD.

**Fig. 5:**
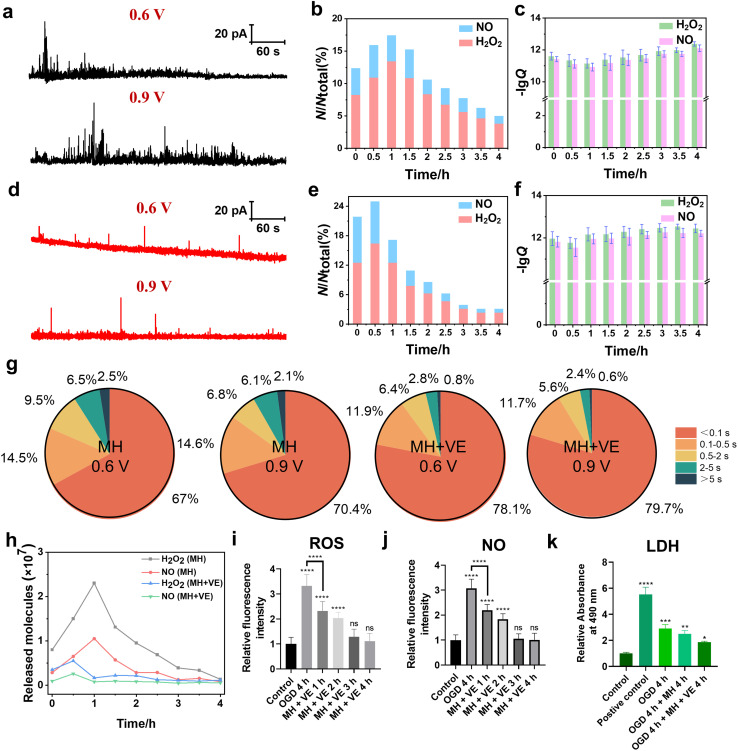
Amperometric traces of H_2_O_2_ and NO effluxes, and cell viability and LDH activities of HT22 cells under MH and VE treatments. (a) Representative amperometric traces at 0.6 V and 0.9 V of HT22 cells under MH for 1 h, respectively. (b) The frequency distributions of the release events of H_2_O_2_ and NO of the MH groups detected at 0.6 and 0.9 V *vs.* time. (c) Statistical analyses of lg *Q* of the MH groups at 0.6 and 0.9 V during 0–4 h, respectively. (d) Representative amperometric traces at 0.6 V and 0.9 V of HT22 cells in MH with VE treatment for 0.5 h. (e) The frequency distributions of the release events of H_2_O_2_ and NO of the MH with VE groups detected at 0.6 and 0.9 V *vs.* time. (f) Statistical analyses of lg *Q* of the MH with VE groups at 0.6 and 0.9 V during 0–4 h, respectively. (g) Statistical diagram of frequencies of different types of amperometric spikes at 0.6 V and 0.9 V of the MH group and MH with VE treatment group. (h) Average released molecules of H_2_O_2_ and NO during 0–4 h of the MH group and MH with VE treatment group. (i and j) Statistical analyses of intracellular H_2_O_2_ and NO levels of HT22 cells during 4 h of OGD (*n* = 30). (k) Quantified LDH activities of HT22 cells using LDH assay (*n* = 3). All data are depicted as means ± SEM and graphs were compared by one-way ANOVA (ns, no significant difference, *p < 0.05, **p < 0.01, ***p < 0.001 and ****p < 0.0001).

As shown in Fig. 5a and b, the amperometric traces of H_2_O_2_ and NO effluxes of HT22 cells under MH for 1 h exhibited a large amount of release events with a maximum frequency distribution ratio of H_2_O_2_ and NO effluxes of 7.3 : 2.7. This can be possibly attributed to the reoxygenation effect triggered by the massive activation of oxygen resupply to induce the overproduction of various oxygen-derived free radicals within 1 h.^46,47^ The calculated charges of the amperometric spikes at 0–4 h of MH showed that the average released *N*_H_2_O_2__ was 0.80 × 10^7^, 2.3 × 10^7^, 1.0 × 10^7^, 0.39 × 10^7^ and 0.14 × 10^7^ molecules, while *N*_NO_ was 0.29 × 10^7^, 1.1 × 10^7^, 0.29 × 10^7^, 0.13 × 10^7^ and 0.08 × 10^7^ molecules, respectively (Fig. 5c). These results reveal that the H_2_O_2_ and NO effluxes initially increased within the first hour of MH, followed by a continuous decrease, indicating that MH can reduce the oxidative stress due to reoxygenation after OGD.^48^ Additionally, the fluorescence results of both intracellular ROS and NO levels of HT22 cells under MH showed no obvious difference in the MH groups until 4 h compared to the relative fluorescence intensities of ROS and NO of the control groups (Fig. S6†). And the viability of HT22 cells improved to 75% after 4 h of MH (Fig. S7†), indicating that MH can inhibit the overproduction of ROS and NO of HT22 cells after suffering from OGD. But the efficiency in alleviating cellular oxidative stress and improving cell viability was still limited. Therefore, to substantially reduce the degree of oxidative stress and improve cell survival, it is necessary to combine MH with other neuroprotective strategies to achieve more comprehensive neuroprotective effects.

To further mitigate the oxidative injury of HT22 cells induced by OGD, we added vitamin E (VE), an antioxidant regularly used in clinics, into the cell culture and evaluated the synergistic therapeutic effect of MH treatment with VE. First, to check whether the addition of VE affects our electrochemical detections of H_2_O_2_ and NO, we recorded the cyclic voltammogram in the advanced Tyrode's solution after adding VE in the potential range of 0 to 1 V. From Fig. S8,† we can see that there was no oxidation current signal in the potential range of 0.6 to 0.9 V, confirming that VE did not interfere with our electrochemical detection of H_2_O_2_ and NO. Then, we added VE into the cell culture medium after MH treatment for 4 h. As shown in Fig. 5d and e, the release events and the frequency distributions of H_2_O_2_ and NO effluxes of HT22 cells reached a maximum after 0.5 h of MH combined with VE treatment, indicating the more effective decreases in the released H_2_O_2_ and NO effluxes compared to the MH groups (Fig. 5a and b). These results indicate that the synergetic therapeutic effect of MH and VE treatment lead to a more rapid and effective reduction of neuronal oxidative stress levels compared to the MH treatment alone. Additionally, the average release of *N*_H_2_O_2__ and *N*_NO_ from HT22 cells presented a maximum of 0.56 × 10^7^ and 0.26 × 10^7^ molecules, respectively, under 0.5 h of MH with VE treatment (Fig. 5f), which were much lower than the *N*_H_2_O_2__ = 1.5 × 10^7^ and *N*_NO_ = 0.65 × 10^7^ molecules of the MH group at 0.5 h (Fig. 5c). This indicates that the combination of MH with VE treatment can effectively reduce the number of released molecules of H_2_O_2_ and NO with a significant decrease in 0.5 h.

Moreover, 90.0% and 91.4% of the amperometric spikes at 0.6 V and 0.9 V were shown to be simple events after addition of VE, suggesting that the oxidative burst in the cells was significantly suppressed with addition of VE (Fig. 5g). And the variation of *N*_H_2_O_2__ and *N*_NO_ during 4 h of MH with VE treatment showed a significant decrement compared to the MH groups (Fig. 5h). The fluorescence results of the ROS and NO effluxes also showed significant decreases compared to the OGD groups. And there was an obvious difference in the group of MH with VE treatment for 1 h compared to the OGD group, demonstrating the rapid and effective scavenging of the released ROS and NO from HT22 cells (Fig. 5i, j and S9†). These results suggest that the oxidative burst of HT22 cells suffering from OGD with reoxygenation is vital for neuronal oxidative injury, and the synergistic effect of MH and VE treatment can significantly ameliorate the oxidative stress levels of HT22 cells from reoxygenation within 1 h.

Next, we further evaluated the therapeutic effect of VE through cell viability and lactate dehydrogenase (LDH) assay experiments. As shown in Fig. S7,† after 4 h of MH with VE treatment, the cell viability was restored to 87.7%, while the extracellular LDH level presented a minimal difference compared to the control group (Fig. 5k), suggesting that the lipid peroxidation injury of HT22 cells was caused by the increased oxidative stress under OGD and thus the degradation of the cell membrane.^49,50^ The lipophilic VE inhibited the subsequent free radical attack and thus protected the cell membrane.^51^ These results demonstrate that the MH treatment with antioxidants can significantly improve neuronal survival, and the OGD-induced oxidative stress may be a key factor for the reduction of cellular viability.

## Conclusion

In this work, we constructed an atmosphere- and temperature-controlled SECM platform to *in situ* monitor the released H_2_O_2_ and NO of HT22 cells under OGD and MH treatment alongside the antioxidant intervention. The SECM results showed the rapid and dramatic release of H_2_O_2_ and NO within 2 h of OGD, while the rates of oxidative stress slowed down during 2–4 h due to the insufficient amount of oxygen, indicating that the neuronal oxidative stress was mainly manifested through ROS release during OGD. The MH treatment partially inhibited the reoxygenation-induced ROS and NO overproduction, which quickly reduced the frequencies and amounts of H_2_O_2_ and NO effluxes with the synergistic effect of MH treatment with antioxidant intervention. Our results reveal that the rapid overproduction of ROS and NO of neurons under OGD can impair the redox balance of the cellular antioxidant system, which causes oxidative stress and influences the integrity of the cell membrane through lipid peroxidation, and even results in the loss of cell viability. The synergetic effect of MH and antioxidant intervention can effectively reduce the generation of cellular ROS and NO and mitigate the associated cellular damage. Our work demonstrates that the oxidative stress of HT22 cells under OGD and reperfusion is the primary cause of neuronal injury and highlights the protective effect of the combination of MH treatment with antioxidants, contributing to a better understanding of the pathophysiology of oxidative stress in cerebral ischemia and offering reference for strategies for clinical management of stroke-related conditions. The developed SECM platform equipped with temperature- and atmosphere-controlled functions can also be used as a versatile tool for *in situ* monitoring the cellular released substances *in vitro* for study of heat and hypoxia-related diseases.

## Data availability

Experimental details (including chemicals and materials, fluorescent staining of ROS and NO levels, cytotoxicity assay and LDH leakage assay of HT22 cells during OGD with MH and with/without VE treatment, preparation of SECM probes, amperometric traces of H_2_O_2_ and NO released from HT22 cells after the addition of DMNQ and L-Arg, and linear sweep voltammograms of VE in advanced Tyrode's solution), the parameters of the SECM theoretical model and the supporting experimental data are all provided in the ESI.

## Author contributions

J. J. Z., F. X. and F. L. conceived the ideas and designed the experiments. F. L. directed the cell and SECM experiments. J. J. Z., Y. L. L., Y. X. Z. and S. Y. Z. conducted the experiments and analyzed the data. All authors interpreted data and contributed to the writing of the manuscript.

## Conflicts of interest

The authors declare no conflict of interest.

## Supplementary Material

SC-015-D4SC05977H-s001
