# Supplementary material for: Synergetic effect of mild hypothermia and antioxidant treatment on ROS–mediated neuron injury under oxygen–glucose deprivation investigated by scanning electrochemical microscopy
Source: Chem Sci. 2024 Nov 12;15(48):20177–88. doi: 10.1039/d4sc05977h (PMC11575619; doi:10.1039/d4sc05977h)
Supplement: SC-015-D4SC05977H-s001 [file SC-015-D4SC05977H-s001.pdf]

Supplementary Information (SI) for Chemical Science.
This journal is © The Royal Society of Chemistry 2024

Electronic Supplementary Information

Synergetic effect of mild hypothermia and antioxidant treatment on ROS-mediated neuron injury under oxygen-glucose deprivation investigated by scanning electrochemical microscopy

Junjie Zhang,[a,b] Yulin Liu,[a,b] Yuxiang Zhao,[a,b] Siyu Zhang,[a,b] Feng Xu[a,b] and Fei Li*[a,b]

[a] *The Key Laboratory of Biomedical Information Engineering of Ministry of Education, School of Life Science and Technology, Xi'an Jiaotong University, Xi'an 710049, P. R. China*

[b] *Bioinspired Engineering and Biomechanics Center (BEBC), Xi'an Jiaotong University, Xi'an 710049, P. R. China*

**Corresponding author: feili@mail.xjtu.edu.cn*

Table of Contents

1.Experimental section S3
1.1 Chemicals and materials S3
1.2 Cell culture S3
1.3 Cytotoxicity assay S3
1.4 Cell staining S4
1.5 LDH leakage assay S4
1.6 Preparation of NO solution S4
1.7 Selectivity and feasibility experiments S4
1.8 SECM experiments S5
1.9 Quantitative analysis of H_2O_2 and NO effluxes S5
1.10 Statistical analysis S6
2.SECM theoretical model S6
Table S1. Main experimental parameters used in the SECM 2D simulation model S7
3.Supporting experimental data S8
Fig. S1 Fluorescence images of intracellular ROS and NO levels of HT22 cells under OGD and MH for 0-8 h. S8
Fig. S2 Temperature-time curves of the actual and setting temperatures at 32 and 37℃ S8
Fig. S3 Amperometric traces of H_2O_2 and NO released from HT22 cells after the addition of DMNQ and L-Arg. S9
Fig. S4 Schematic diagram of the SECM theoretical model and concentration maps of NO efflux released from cell membrane boundary using a 10-50 μm microelectrode at 32℃ and 37℃. S9
Fig. S5 Fluorescence images and statistic results of intracellular ROS and NO levels of HT22 cells under OGD for 0-4 h. S9
Fig. S6 Fluorescence results of intracellular ROS and NO levels of HT22 cells under OGD with following MH for 0-4 h. S10
Fig. S7 Viability of HT22 cells under OGD and MH with/without VE treatment for 4 h. S10
Fig. S8 Linear sweep voltammogram in advanced Tyrode's solution with and without vitamin E S10
Fig. S9 Fluorescence images of intracellular ROS and NO levels of HT22 cells under OGD with following MH combined with VE treatment for 0-4 h. S11
Notes and references S11

Experimental section

1.1 Chemicals and materials

Magnesium sulfate was purchased from Zhiyuan chemical regent Co., Ltd. (China). Calcium chloride anhydrous and sodium chloride were bought from Tianli chemical regent Co., Ltd. (China). Sodium bicarbonate and vitamin E (VE) were bought from Energy Chemical Technology Co., Ltd. (China). Sodium phosphate monobasic monohydrate was obtained from Aladdin Biochemical Technology Co., Ltd. (China). 2,3-dimethoxy-1,4-naphthalenedione (DMNQ) was purchased from MedChemExpress (USA). Ascorbic acid (AA), glutathione (GSH), L-glutamic acid (Glu), nicotinamide adenine dinucleotide (NADH) and L-arginine (L-Arg) were all bought from Sigma-Aldrich (USA). Potassium chloride (KCl, 99%) and H_2O_2 (30%) were purchased from Sinopharm Chemical Reagent Co., Ltd. (China). Advanced Tyrode's solution was obtained from Procell Co., Ltd. (China). Hoechst 33342 and live/dead kit (L3224) were purchased from Thermo Scientific (U.S.A.). ROS/superoxide detection assay (ab139476) was obtained from Abcam (U.K.). Cell counting Kit-8 (CCK-8) and lactate dehydrogenase (LDH) assay kit were required from Dojindo (Japan). NO fluorescence probe (DAF-FM DA) was ordered from Beyotime Biotechnology (China). High-glucose Dulbecco's modified Eagle medium (HG-DMEM) and fetal bovine serum (FBS) were purchased from Gibco (U.S.A.). Pure nitric oxide (NO) gas was ordered from Shaanxi KuaiTe Gas Equipment Co., Ltd. (China). Deionized water (Millipore, resistivity >18.2 MΩ, U.S.A.) was used to prepare all the aqueous solutions used in this work. The 0.22 μm membranes (Millex-GP, Germany) was employed to filter the solutions before use in cell experiments.

1.2 Cell culture

The hippocampal neuronal line of HT22 cell, was bought from Sigma-Aldrich (SCC129, U. S. A) and cultured in the HG-DMEM added with 10% FBS at 37°C. The medium of HT22 cells was changed to the glucose-free DMEM in a hypoxic incubator (37°C, 1% O_2) in the OGD experiments. The culture medium was replaced with the complete HG-DMEM supplemented under mild hypothermia (MH) condition (32°C, 20% O_2, 5% CO_2).

1.3 Cytotoxicity assay

The viability of HT22 cells cultured in the 96-well microtiter plate (1×10^4 cells/well) was measured using CCK-8 assays. Then the sample wells were filled with CCK-8 solution (10 μL per well) and incubated for 1 h.The absorbance of sample wells at 450 nm was measured using a multifunctional microplate reader (TECAN SPARK 10 M).

1.4 Cell staining

Intracellular ROS and NO levels and of HT22 cells were characterized using the ROS/superoxide detection assay kit and the NO fluorescence probe, respectively. After the OGD and MH treatment, the fluorescent ROS and NO indicators were gently mixed to make a 1:1000 diluted solution, and then the HT22 cells (2×10^5 cells/dish) were labeled under dark at 37°C for 1 h. The nucleus of HT22 cells was stained with Hoechst 33342 during the last 5 min of the 1 h incubation. The HT22 cells were rinsed with PBS for three times and characterized with a confocal microscope (Olympus FV3000). Quantification of the fluorescence intensities of intracellular ROS and NO levels of HT22 cells was performed using ImageJ.

1.5 LDH leakage assay

For measuring the LDH cytotoxicity of HT22 cells, the density of the HT22 cells were set as 1×10^4 cells/well in the 96-well plates. After OGD and MH treatment, the extracellular LDH levels were measured by the LDH assay kit according to the manufacturer' instructions. The fluorescence intensities and absorbances of the culture medium of HT22 cells at 490 nm were recorded by a multifunctional microplate reader (TECAN SPARK 10 M).

1.6 Preparation of NO solution

The saturated NO solution was prepared by the following precedure. First, 100 mL of advanced Tyrode's solution (pH 7.4) was deoxygenated by purging with pure nitrogen gas (N_2) in a sealed glass vessel for 2 h to ensure complete elimination of dissolved oxygen. Then the input N_2 was replaced with pure NO gas, which was introduced into the deoxygenated solution through gentle bubbling. After 1 h of constant bubbling, the solution was saturated with NO with approximate concentration of 1.9 mM at room temperature.[1] Finally, the saturated NO solution was transferred to a sealed three-electrode electrochemical cell for immediate uses for the subsequent electrochemical experiments.

1.7 Selectivity and feasibility experiments

Amperometric measurements were performed with a three-electrode system using a 25 μm-in-diameter Pt microelectrode as the working electrode. In the selectivity experiments, the amperometric measurements were recorded at 0.6 V and 0.9 V in the advanced Tyrode's solution containing 10 μM of H_2O_2 and NO and added with interferents of 100 μM of AA, GSH, Glu, and NADH. In the feasibility experiments, the amperometric measurements were

performed at 0.6 V and at 0.9 V in the advanced Tyrode's solution added with 30 μM DMNQ or 100 μM L-Arg, respectively.

1.8 SECM experiments

The SECM instrument (ElProScan PG618, HEKA Elektronik GmbH, Harvard Bioscience Inc.) installed on an inverted optical fluorescence microscope (Olympus-IX53, Olympus Co., Ltd., Japan) was used for all SECM experiments. SECM measurements were performed using a three-electrode system consisting of a 25 μm-in-diameter Pt microelectrode (*RG* = 3, where *RG* is the ratio of the entire electrode radius to the radius of Pt wire) as the working electrode and the SECM probe, a 0.6 mm-in-diameter Ag/AgCl wire and a 0.5 mm-in-diameter Pt wire as the reference electrode and counter electrode, respectively. To ensure the precise control of the atmospheric condition and temperature during SECM experiments, a semi-enclosed polycarbonate gas chamber integrated with a temperature-controlled incubator was designed and constructed (**Fig. 2a**). The chamber sidewalls were equipped with gas inlet channels, and a clearance at the top of the chamber allowed for the flexible horizontal and vertical movement of the SECM probe, as well as provided a gas outlet channel. The precise ratio of gases under two atmospheric conditions (1% oxygen, 5% CO_2 and 94% N_2 for OGD; 5% CO_2, 20% oxygen and 75% N_2 for physiological culture and MH treatment) was obtained through an automatic gas mixing proportioner (MAP-003, Hoda Micro Control Co., Ltd, China). The culture dish incubator was set at 37.0±0.2°C and 32.0±0.2°C under the OGD and MH treatment, respectively. The glucose-free Earle's balanced salt solution (117.24 mM NaCl, 5.33 mM KCl, 26.19 mM $NaHCO_3$, 1.01 mM NaH_2PO_4, 1.80 mM $CaCl_2$, 1.01 mM NaH_2PO_4) was used in the SECM experiments to mimic the OGD culture model. The medium of HT22 cells was replaced with advanced Tyrode's solution under MH with and without VE treatment. The whole atmosphere- and temperature-controlled SECM platform was placed in a Faraday cage and well grounded. Amperometric measurements were carried out every 30 min under OGD for 4 h and MH with/without VE treatment. The chronoamperometrograms were recorded at 0.60 V and 0.90 V (vs. Ag/AgCl RE) for monitoring the cellular released H_2O_2 and NO, respectively. The peack currents of the amperometric spikes with higher than 3-fold of the standard deviation of background noise were recorded. Amperometric spikes were collected and analyzed by the self-programmed Matlab software.

1.9 Quantitative analysis of H_2O_2 and NO effluxes

The areas under the recored amperometric spikes were integrated according to eq. (S1) to calculate the electrical charge (Q).

$$Q = \int I \mathrm{d}t \qquad \text{(S1)}$$

where I is the current, and t is time. The electrical charges of H_2O_2 and NO were obtained individually based on eqs. (S2) and (S3).

$$Q_{0.6\ \mathrm{V}} = Q_{\mathrm{H_2O_2}} \qquad \text{(S2)}$$

$$Q_{0.9\ \mathrm{V}} = Q_{\mathrm{H_2O_2}} + Q_{\mathrm{NO}} \qquad \text{(S3)}$$

The corresponding charges were converted to the amounts of H_2O_2 and NO based on the Faraday's law (eq. (S4)).

$$n = Q/zF \qquad \text{(S4)}$$

where n is the molar amount of redox analyses, F is the Faraday constant (9.65×10^4 C/mol), and z is the number of transferred electrons (for H_2O_2 oxidation ($H_2O_2 - 2e^- \rightarrow O_2 + 2H^+$), z = 2; for NO oxidation ($NO + 2H_2O - 3e^- \rightarrow NO_3^- + 4H^+$), z = 3)[2, 3]. The number of H_2O_2 and NO molecules were calculated according to eq. (S5).

$$N = nN_A \qquad \text{(S5)}$$

where N_A is the Avogadro constant (6.02×10^{23} mol^{-1}).

1.10 Statistical analysis

Statistical analysis was performed using GraphPad Prism 8 (GraphPad Software, USA). Statistics are presented as the means±SEM for all the quantitative data with $n \geq 3$ for the cell experiments. Statistical analysis between two groups was made using the two-sample t-test. One-way ANOVA was used to analyze multiple groups (*ns*, no significant difference, *$p < 0.05$, **$p < 0.01$, ***$p < 0.001$, and ****$p < 0.0001$).

2 SECM theoretical model

In our work, finite element method (FEM) was used to analyze the theoretical concentration maps and amperometric traces of H_2O_2 and NO effluxes from the cell membrane boundary. In detail, the simulation model of SECM system was established in a two-dimensional axisymmetric coordinate system using COMSOL Multiphysics software 6.0 (COMSOL Inc., Sweden). As shown in **Fig. S4a**, the origin of the coordinate axes was set at the center of the microdisk electrode (RG = 3), and the domains of cell and the bulk solution were defined as two separate domains. The cell membrane was defined as an outward flux boundary from the cell to the solution domain. The diffusions of H_2O_2 and NO in the solution domain follow the Fick's second law, which can be expressed by eqn. (S6).

$$\frac{\partial CA(r,z,t)}{\partial t} = D_A\left(\frac{\partial^2 CA(r,z,t)}{\partial r^2} + \frac{1\partial CA(r,z,t)}{r\quad \partial r} + \frac{\partial^2 CA(r,z,t)}{\partial z^2}\right) \tag{S6}$$

where *r* and *z* are the axisymmetric coordinates in the model, *t* is the time, and *D* represents the diffusion coefficient of H_2O_2 or NO in the advanced Tyrode's solution, respectively.

The concentration distribution map was obtained by integrating the flux to the probe tip at each simulated probe position based on the oxidation currents of H_2O_2 and NO on the SECM probe tip at 0.6 V and 0.9 V, respectively. And the current at the SECM probe tip can be calculated by eqn. (S7).

$$i = 2\pi nD\mathrm{F}\int_0^{\mathrm{a}} \mathrm{r}\left[\frac{\partial C_{\mathrm{A}}(r,z,t)}{\partial z}\right]\mathrm{dr} \tag{S7}$$

where i depends on the concentration gradient ($\partial C_A/\partial z$) of H_2O_2 and NO, n is the number of electrons transferred in the redox reaction (n = 2 for H_2O_2, n = 3 for NO). The main experimental parameters used in the SECM 2D simulation model are listed in **Table S1**.

Table S1. Main experimental parameters used in the SECM 2D simulation model

Parameter	Unit	Value
a	μm	5.0, 12.5, 25.0
RG	-	3.0
$C_{H_2O_2}$	μmol	1
C_{NO}	μmol	1
T	K	305.0, 310.0
$D_{H_2O_2}$	cm^{-2} s^{-1}	1.92×10^{-5} (32°C)<br>2.17×10^{-5} (37°C)[4]
D_{NO}	cm^{-2} s^{-1}	3.94×10^{-5} (32°C)<br>4.92×10^{-5} (37°C)[5]
h_{cell}	μm	6.0
r_{cell}	μm	12.0

3. Supporting experimental data

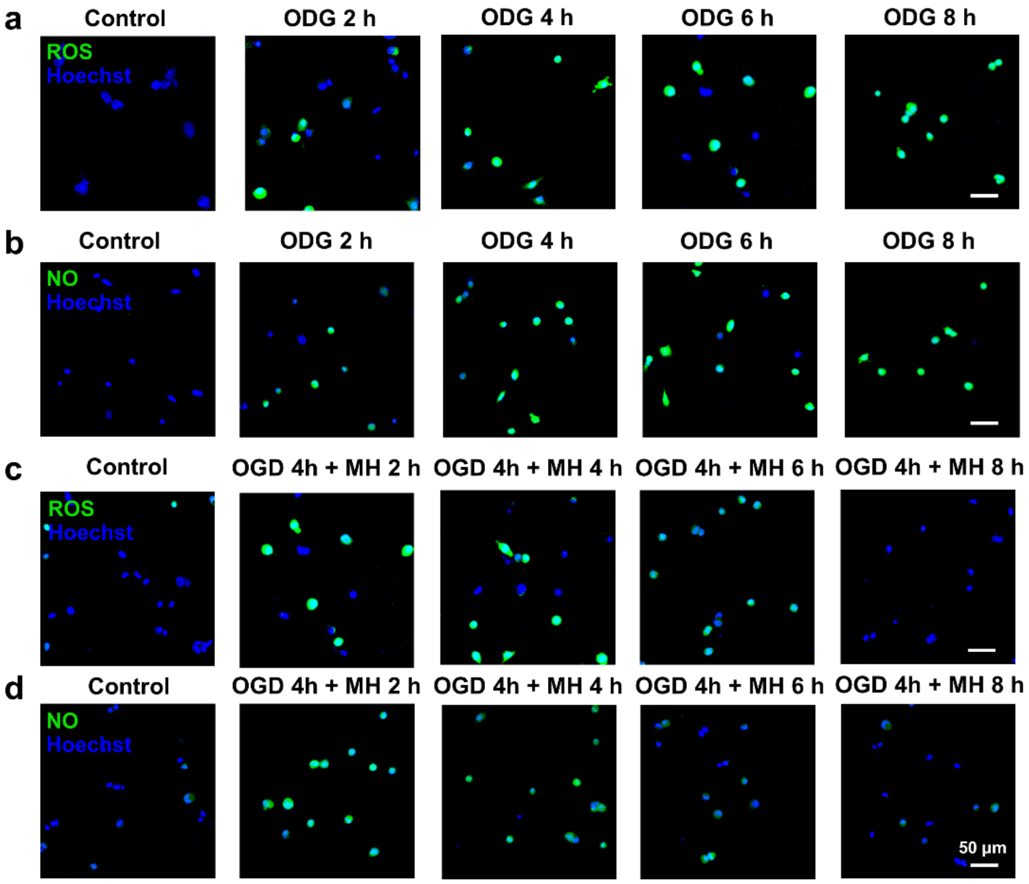

Fig. S1 Fluorescence images of the intracellular ROS and NO levels of HT22 cells under (a and b) OGD and (c and d) MH treatment for 0-8 h.

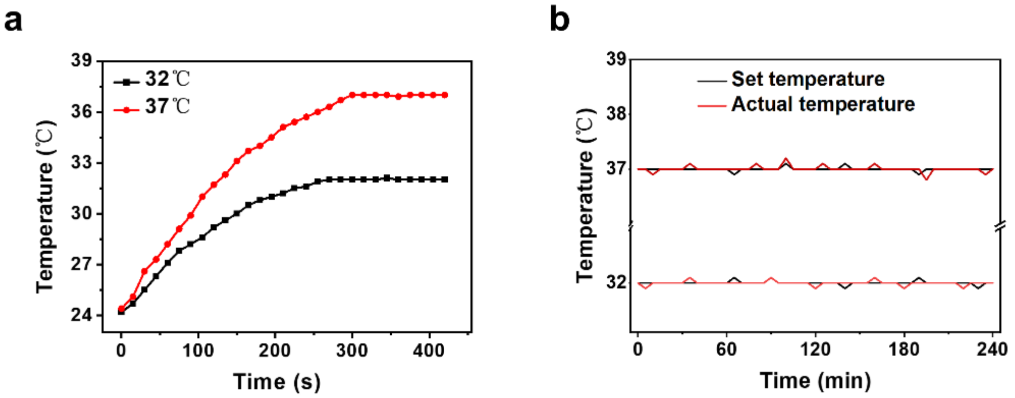

Fig. S2 (a) Heating curves and (b) temperature-time curves of the actual and setting temperatures at 32 and 37°C in our SECM experimental system.

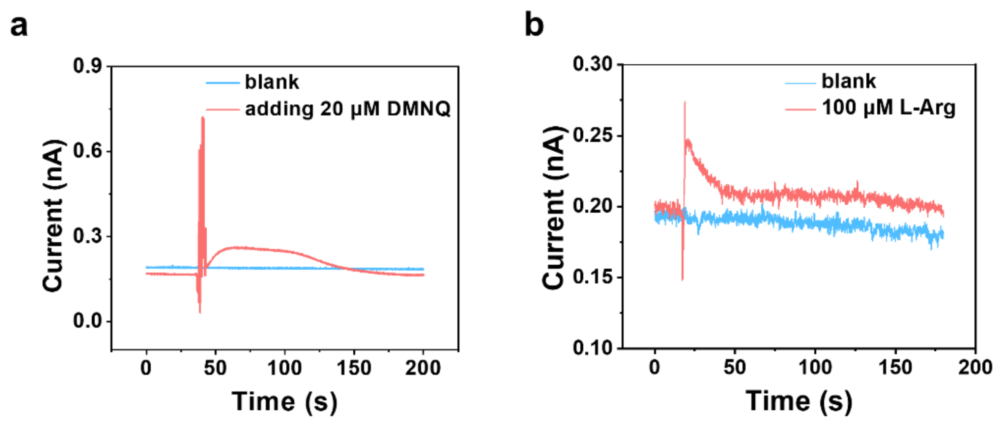

Fig. S3 Amperometric traces of (a) H_2O_2 at 0.6 V and (b) NO at 0.9 V released from HT22 cells after additions of DMNQ and L-Arg in the advanced Tyrode's solution.

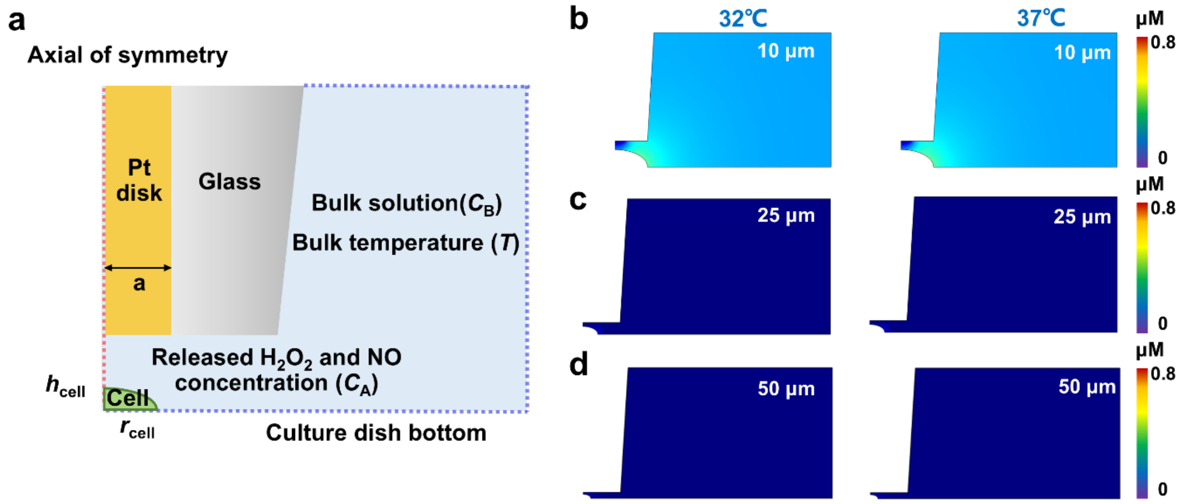

Fig. S4 Schematic diagram of (a) SECM theoretical model and (b-d) concentration maps of NO effluxes released from the cell membrane using (a, b) 10, (c, d) 25 and (e, f) 50 μm-in-diameter Pt microdisk electrodes at 32°C and 37°C.

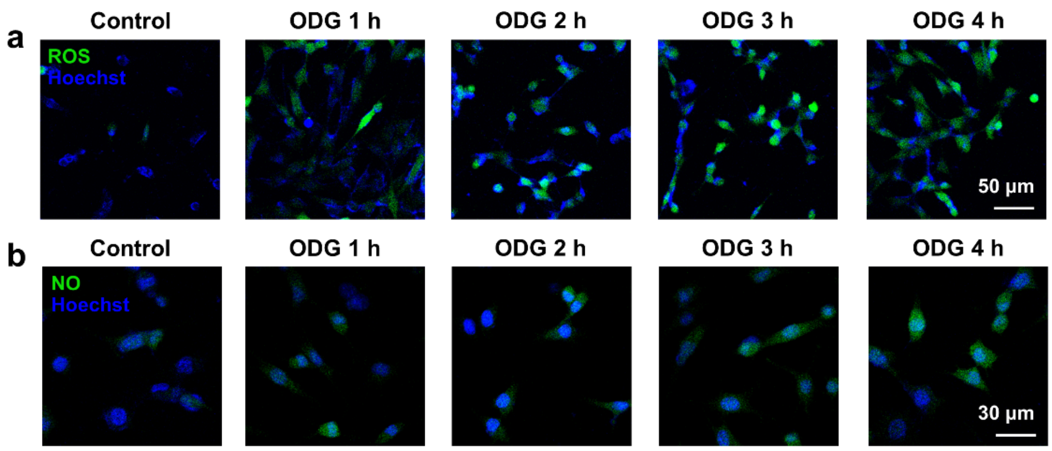

Fig. S5 Fluorescence images and statistic results of the intracellular (a) ROS and (b) NO levels of HT22 cells under OGD for 1-4 h.

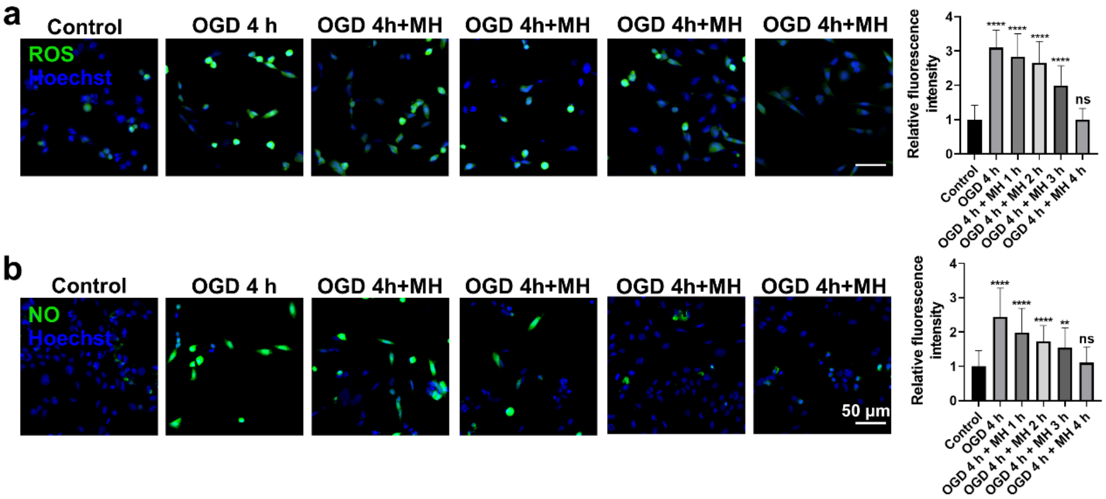

Fig. S6 Fluorescence results of the intracellular (a) ROS and (b) NO levels of HT22 cells under OGD after MH treatment for 0-4 h. All data are depicted as means±SEM, and graphs were compared by one-way ANOVA (*ns*, no significant difference, ***p*<0.01 and *****p*<0.0001).

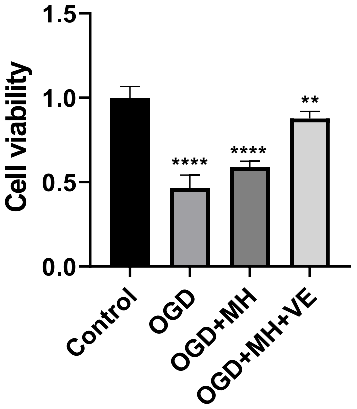

Fig. S7 Viability of HT22 cells under OGD and MH treatment with/without adding VE for 4 h. All data are depicted as means±SEM, and graphs were compared by one-way ANOVA (***p*<0.01 and *****p*<0.0001).

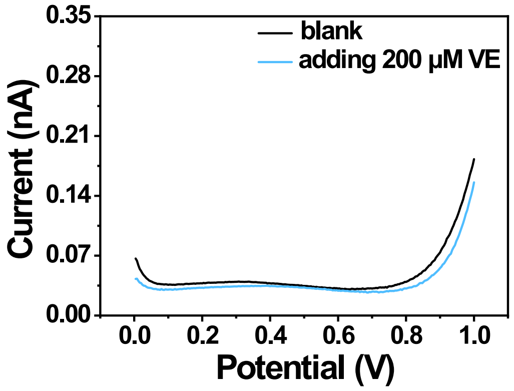

Fig. S8 Differential pulse voltammogram in the advanced Tyrode's solution without (black curve) and with (blue curve) adding 200 µM VE, respectively.

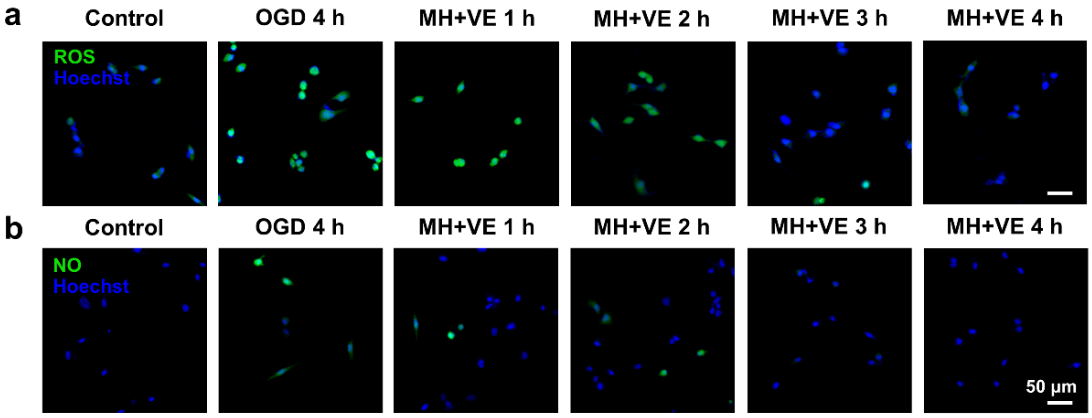

Fig. S9 Fluorescence images of the intracellular (a) ROS and (b) NO levels of HT22 cells under OGD after MH with VE treatment for 0-4 h.

Notes and references

1. J. Cancino, S. Borgmann, S. A. S. Machado, V. Zucolotto, W. Schuhmann and J. Masa, Electrochemical sensor for nitric oxide using layered films composed of a polycationic dendrimer and nickel(II) phthalocyaninetetrasulfonate deposited on a carbon fiber electrode, *Microchim. Acta*, 2015, **182**, 1079-1087.
2. B. J. Privett, J. H. Shin and M. H. Schoenfisch, Electrochemical nitric oxide sensors for physiological measurements, *Chem. Soc. Rev.*, 2010, **39**, 1925.
3. W.-T. Fan, Y. Zhao, W.-T. Wu, Y. Qin, J. Yan, Y.-L. Liu and W.-H. Huang, Redox homeostasis alteration in Endothelial mechanotransduction monitored by dual stretchable electrochemical sensors, *Anal. Chem.*, 2022, **94**, 7425-7432.
4. J. Zhang, Y. Liu, Y. Li, T. Zhu, J. Qiu, F. Xu, H. Zhang and F. Li, In situ and quantitatively imaging of heat-induced oxidative state and oxidative damage of living neurons using scanning electrochemical microscopy, *Small Methods*, 2022, **6**, 2200689.
5. D. L. Wise and G. Houghton, Diffusion coefficients of neon, krypton, xenon, carbon monoxide and nitric oxide in water at 10–60°C, *Chem. Eng. Sci.*, 1968, **23**, 1211-1216.
